# Gene Signature Associated With Bromodomain Genes Predicts the Prognosis of Kidney Renal Clear Cell Carcinoma

**DOI:** 10.3389/fgene.2021.643935

**Published:** 2021-06-02

**Authors:** Junwan Lu, Changrui Qian, Yongan Ji, Qiyu Bao, Bin Lu

**Affiliations:** ^1^Protein Quality Control and Diseases Laboratory, Key Laboratory of Medical Genetics of Zhejiang Province, Key Laboratory of Laboratory Medicine, Ministry of Education of China, School of Laboratory Medicine and Life Sciences, Wenzhou Medical University, Wenzhou, China; ^2^Wenzhou Central Hospital, Wenzhou, China

**Keywords:** KIRC, bromodomain genes, prognosis signatures, overall survival, TCGA

## Abstract

Bromodomain (BRD) proteins exhibit a variety of activities, such as histone modification, transcription factor recruitment, chromatin remodeling, and mediator or enhancer complex assembly, that affect transcription initiation and elongation. These proteins also participate in epigenetic regulation. Although specific epigenetic regulation plays an important role in the occurrence and development of cancer, the characteristics of the BRD family in renal clear cell carcinoma (KIRC) have not been determined. In this study, we investigated the expression of BRD family genes in KIRC at the transcriptome level and examined the relationship of the expression of these genes with patient overall survival. mRNA levels of tumor tissues and adjacent tissues were extracted from The Cancer Genome Atlas (TCGA) database. Seven BRD genes (*KAT2A, KAT2B, SP140, BRD9, BRPF3, SMARCA2*, and *EP300*) were searched by using LASSO Cox regression and the model with prognostic risk integration. The patients were divided into two groups: high risk and low risk. The combined analysis of these seven BRD genes showed a significant association with the high-risk groups and lower overall survival (OS). This analysis demonstrated that total survival could be predicted well in the low-risk group according to the time-dependent receiver operating characteristic (ROC) curve. The prognosis was determined to be consistent with that obtained using an independent dataset from TCGA. The relevant biological functions were identified using Gene Set Enrichment Analysis (GSEA). In summary, this study provides an optimized survival prediction model and promising data resources for further research investigating the role of the expression of BRD genes in KIRC.

## Introduction

The latest database estimates that as incidence rates have doubled in the last several decades, renal carcinoma (RCC) will be the eighth most common tumor in the United States with approximately 70,000 new cases expected in the United States alone and more than 300,000 new cases expected globally by 2020 (Motzer et al., 2017; Bacigalupa and Rathmell, 2020). Clear cell carcinoma of the kidney (KIRC, 70–75%), renal papillary cell carcinoma (KIRP, 10–16%), and chromophobe of the kidney (KICH, approximately 5%) represent greater than 90% of RCC cases (Shuch et al., 2015).

In 1992, Tamkun et al. (1992) reported the bromodomain BRD for the first time in a study of Brahma (a *Drosophila* gene) and the female sterility homologous protein. Bromodomains (BRDs) are protein modules consisting of approximately 110 amino acids that recognize acetylated lysine in histones and other proteins (Cochran et al., 2019). BRDs are widely found in proteins with a variety of catalytic and scaffold functions and coexist in most tissues (Fujisawa and Filippakopoulos, 2017). These BRD-containing proteins play a number of roles within the cell, but the unique ability of the bromodomain to bind to acetyl-lysine is required for the function of each protein in some capacity. Protein families with bromodomains contain HATs and HAT associated proteins(GCN5L2, general control of amino acid synthesis protein 5-like 2), P300/CBP-associated factor (PCAF, also known as KAT2B), histone methyltransferases (ASH1 L, absent small and homeotic disk protein 1 homolog), bromodomain-containing protein 9 (BRD9), transcriptional regulators (BET, bromodomain and extra-terminal), chromatin-remodeling factors (SMARCA2 SWI/SNF-related matrix-associated actin-dependent regulator of chromatin a2), ATP-dependent chromatin-remodeling complexes (BAZ1B, BRD adjacent to zinc finger domain protein), and transcriptional coactivators (TRIM28 (tripartite motif-containing 28), TAFs) (Zhang et al., 2015). The human proteome encodes 61 BRD modules that are identified in 42 different proteins. BRD-containing proteins have a wide range of documented roles in cellular homeostasis. These proteins participate in histone modification and regulate the recruitment and segregation of transcriptional machinery components to particular loci in normal tissues (Fujisawa and Filippakopoulos, 2017). These proteins have been implicated in human diseases, including neurological and inflammatory diseases, as well as cancers (Muller et al., 2011; Belkina and Denis, 2012; Wang and Filippakopoulos, 2015). BRD-containing proteins are deregulated in cancer cells, and their abnormal expression in various tumors has been shown to both stimulate and inhibit malignant phenotypes. These dual roles of certain BRD-containing proteins (such as TRIM24) in tumor promotion and suppression suggest that they have context-dependent functions, which makes their study intriguing (Fujisawa and Filippakopoulos, 2017).

Bromodomains play important roles in the occurrence and development of cancer. Current studies have mostly focused on the bromodomain and extra-terminal domain (BET) protein family and CREBBP/EP300 (Breen and Mapp, 2018). Bromodomain-containing protein 4 (BRD4), the most famous member of the BET family, is overexpressed in acute myeloid leukemia (AML) cells and regulates the transcription of genes related to AML pathogenesis (Baragano Raneros et al., 2021). Targeting BRD4 by genetic knockdown or chemical inhibitors blocked mitochondrial fission and caused cancer stem cell (CSC) exhaustion and loss of tumorigenic capability (Civenni et al., 2019). Studies have also found that the majority of acute myeloid leukemia patients overexpress CREBBP, and this overexpression is correlated with poor prognosis (Veazey et al., 2020). Similarly, CREBBP is highly expressed in breast cancer patients, and CREBBP is involved in the regulation of cellular events in advanced prostate cancer (Furlan et al., 2021; Ramadan et al., 2021). Although the roles of the BRD family of proteins in some tumors have been revealed, the characteristics of the BRD family in renal clear cell carcinoma (KIRC) have not been determined.

The objective of this research was to investigate the expression levels of BRD genes in renal clear cell carcinoma and determine their relationships with patient survival. As a result, we found that the expression of BRD genes differs between KIRC and adjacent tissues, and some of the BRD genes are related to poor progression and overall survival.

## Materials and Methods

### Data Collection and Processing

Bromodomain gene expression, somatic mutation, copy number and clinical data of kidney renal clear cell cancer were downloaded from TCGA^1^. The following criteria were used to select the patients for model building: (a) histological diagnosis of KIRC; (b) available BRD gene expression and clinicopathological and follow-up data; and (c) an OS of > 30 and < 3,650 days. A total of 515 patients met the requirements and were recruited for further analysis. The expression profiles were normalized using the R package edgeR (Robinson et al., 2010), and genes with an average count > 1 across all patients were retained. The microarray dataset (GSE22541) was downloaded from the Gene Expression Omnibus to validate the model’s prediction of prognosis. The immunohistochemistry marker results were retrieved from The Human Protein Atlas (HPA)^2^. Because all the data were downloaded from public databases, it was not necessary to acquire additional ethical approval for this study.

### Construction of a Potential Prognostic Signature

First, univariable Cox analysis was conducted in the training set to collect BRD genes that were significantly (*P* < 0.05) correlated with the OS probability of KIRC tumor cases. Subsequently, LASSO Cox regression with 10 cross-validations was applied to those significant genes, and potential KIRC prognosis signatures based on these BRD genes were developed. The risk score for each patient was calculated by a standard formula, which combines the expression levels of the mRNAs and LASSO Cox regression coefficients (λs). Patients were then divided into low-risk or high-risk groups according to the median risk score cutoff value. The results (the area under the curve, AUC) of the time-dependent ROC analysis were used to measure prognostic performance. The risk score accuracy was assessed, and OS at 1, 3, and 5 years was predicted. To assess the independence of a BRD gene signature as a predictor, univariate and multivariate Cox regression analyses were used to analyze risk scores and other clinical data, including AJCC stage, tumor grade, sex, and age, at diagnosis. ROC and survival curves were obtained using the program packages “survivalROC” (Heagerty and Zeger, 2000), “survminer” (Kassambara et al., 2017) and “survival” (Nativ et al., 1994).

### Nomogram Analysis

The R package “rms” was used to perform nomogram analysis by including those factors that were significantly associated with the OS of KIRC patients in multivariate analysis. A calibration plot was applied to estimate the discrimination between actual and nomogram-predicted OS probability.

### Pan-Cancer Analysis

The “Gene_DE” module of the TIMER2 (Li et al., 2020) web server was used to explore the differential expression between tumor and adjacent normal tissues for markers across all TCGA tumors. The “Gene_Outcome” module was used to evaluate the clinical relevance of gene expression across various cancer types.

### Statistical Analysis

R software 4.0 was used for all statistical analyses. Two groups of violin plots were analyzed using the Wilcoxon test. The heatmap of the BRD gene was visualized using the R package “pheatmap”. The Kaplan-Meier method was used to compare the OS of the patients in the high− and low-risk groups. Correlations among the BRD genes were assessed by Pearson’s test. GESA analysis was performed using GSEA v4.0 software^3^.

## Results

### Differential Expression of BRD Genes in KIRC and Normal Tissues

**FIGURE 1:**
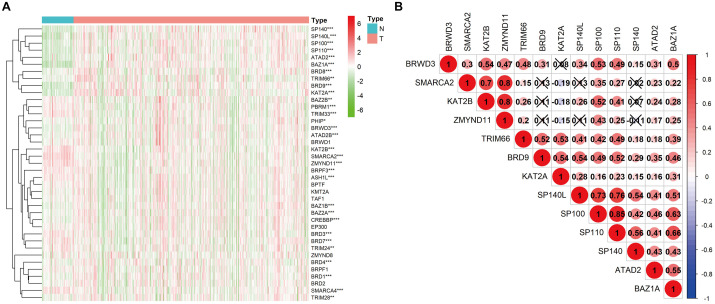
Heatmap and correlation matrix of BRD genes. **(A)** mRNA levels of 42 BRD genes in KIRC. *P < 0.05; **P < 0.01; ***P < 0.001; N, normal control; T, tumor samples; the gene upregulated in red; the gene downregulated in green. **(B)** Correlation between differentially expressed BRD genes.

**FIGURE 2:**
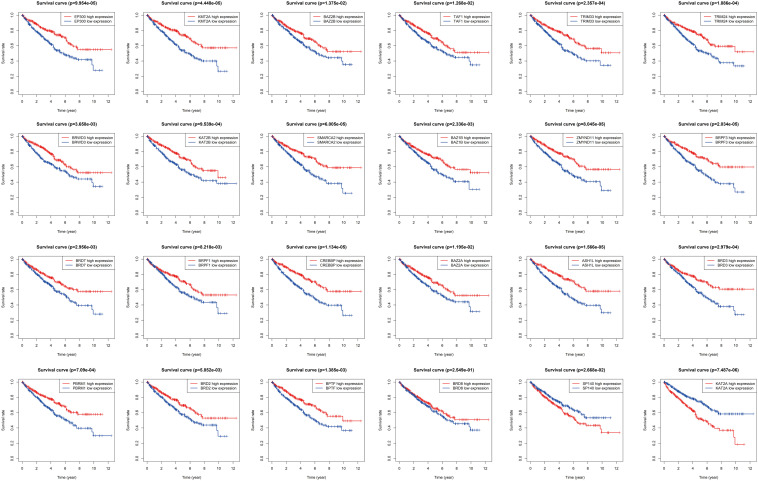
Kaplan-Meier survival analysis for the high and low expression of BRD genes.

Transcriptome data of 535 KIRC samples and 72 paratumor normal tissue samples were downloaded from the TCGA database, and the expression profiles of 42 BRD genes were extracted. Thirty differentially expressed (*P* < 0.01) BRD genes were identified (18 upregulated genes and 12 downregulated genes), among which *SP140*, *BAZ1A*, and *KAT2A* exhibited significantly higher (fold change > 2) expression patterns (Figure 1A and Supplementary Table 1). Furthermore, we analyzed the Spearman correlation among the significantly differentially expressed (log_2_fold change > 0.5) BRD genes (Figure 1B). The expression levels of most genes were positively correlated. The expression of *SP110* was significantly correlated with *SP100* and *SP140L* (cor > 0.75). Moreover, the expression levels of *SMARCA2*, *KAT2B* and *ZMYND11* were highly correlated with each other. The results of Kaplan–Meier analysis showed that 24 of the 42 BRD genes were correlated with KIRC prognosis, and the low expression group had shorter OS than the high expression group for most of these BRD genes except SP140 and KAT2A (Figure 2).

### CNVs and SNPs of the BRD Genes in KIRC Patients

**FIGURE 3:**
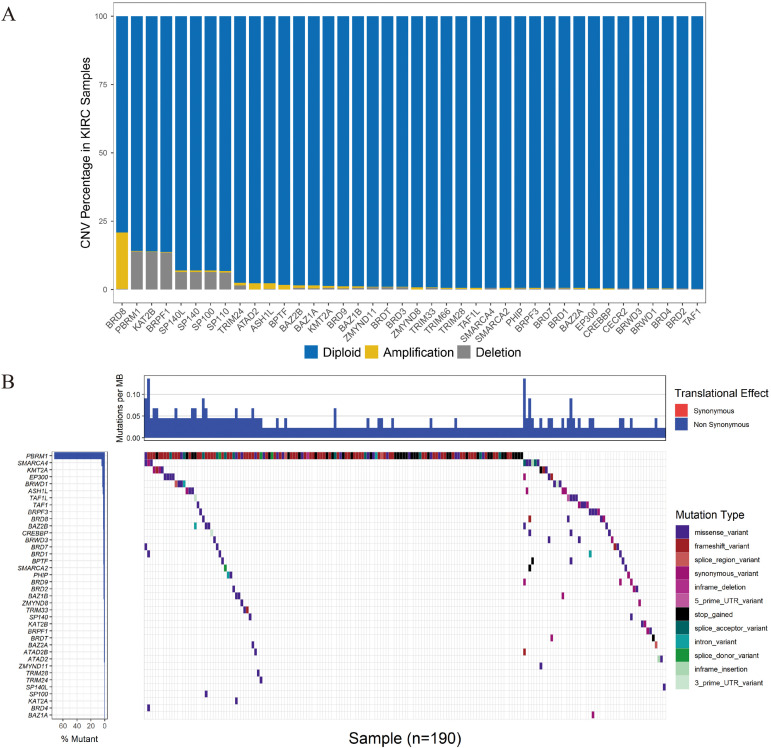
CNV and SNP analysis of the seven BRD genes in the signature. **(A)** The percentage of patients with CNVs. **(B)** SNPs of the BRD genes were observed in 336 patients.

CNV events were frequently observed in the 532 KIRC samples with CNV data (Figure 3A). Specifically, the *BRD8* gene had the highest frequency of CNV events (20.86%, 111/532) followed by *PBRM1* (14.10%, 75/532). The most common CNV patterns of *BRD8* and *PBRM1* were amplification and deletion, respectively. The correlation between the mRNA level and the copy number of the BRD gene with a high CNV rate was systematically evaluated (Supplementary Figure 1). The results showed that the 10 genes (*SP100*, *BAZ2B*, *BAZ1A*, *BRPF3*, *SMARCA2*, *TRIM28*, *TRIM24*, *BRD8*, *BRD7* and *BRD2*) with higher copy numbers showed higher expression levels. Among the 336 KIRC cases with sequencing data, SNPs of BRD genes were identified in 190 independent samples (Figure 3B). The PBRM1 gene had the highest mutation frequency (45.83%, 154/336) followed by *SMARCA* (2.68%, 9/336).

### Construction of the BRD Gene Prognostic Risk Signature

**FIGURE 4:**
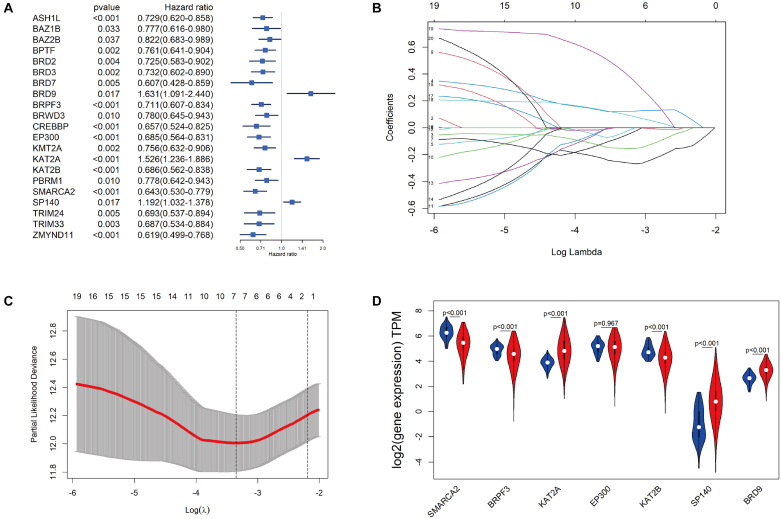
Construction of the BRD gene prognostic risk signature. **(A)** Univariate Cox regression of BRD genes with P-values < 0.05. **(B)** The LASSO coefficient profile of the remaining 21 BRD genes after analysis with univariate Cox regression. **(C)** The partial likelihood deviance is shown against log (lambda) with the vertical line drawn according to the selected values using 10-fold cross-validation. **(D)** Differentially expressed analysis of seven model BRD genes. The normal control is presented in blue, and KIRC samples are presented in red.

The 515 KIRC samples of TCGA were grouped randomly into two cohorts, including a training set (360) and a testing set (155), as shown in Table 1. No significant differences in the clinical and baseline demographic characteristics between the two groups were identified (*P* > 0.05). To define the association of the BRD genes with KIRC overall survival, univariable Cox regression analysis was performed in the testing set, and 21 BRD genes were significantly associated (*P* < 0.05) with the OS of patients (Figure 4A). Furthermore, LASSO Cox regression was performed among these 21 BRD genes (Figures 4B,C), and a seven-BRD gene risk signature was constructed. Risk scores of patients were calculated as follows: risk score = (−0.2545 × expression of *SMARCA2*) + (−0.1522 × expression of *BRPF3*) + (0.1213 × expression of *KAT2A*) + (−0.0445 × expression of *EP300*) + (−0.0714 × expression of *KAT2B*) + (0.1056 × expression of SP140) + (0.4356 × expression of *BRD9*). Different expression levels of the seven genes between tumor and non-tumor tissues were validated. In the KIRC tissues, *KAT2A*, *SP140*, and *BRD9* mRNA levels were upregulated significantly, whereas *SMARCA2*, *BRPF3* and *KAT2B* levels were downregulated significantly (Figure 4D).

**TABLE 1:** Clinicopathological characteristics of included KIRC patients from TCGA database.

		Training set (*n* = 360)	Testing set (*n* = 155)	*P*
Statue	Alive	239	105	0.844
	Dead	121	50	
Age	≤ 50	76	35	0.799
	> 50	284	120	
Gender	Male	235	102	0.988
	Female	125	53	
Stage	Stages I–II	217	94	0.999
	Stages III–IV	143	61	
M	M0	288	124	1
	M1-MX	72	31	
N	N0	166	69	0.813
	N1-NX	194	86	
T	T1-T2	231	98	0.917
	T3-T4	129	57	
Grade	G1-G2	169	68	0.585
	G3-G4	191	87	

**FIGURE 5:**
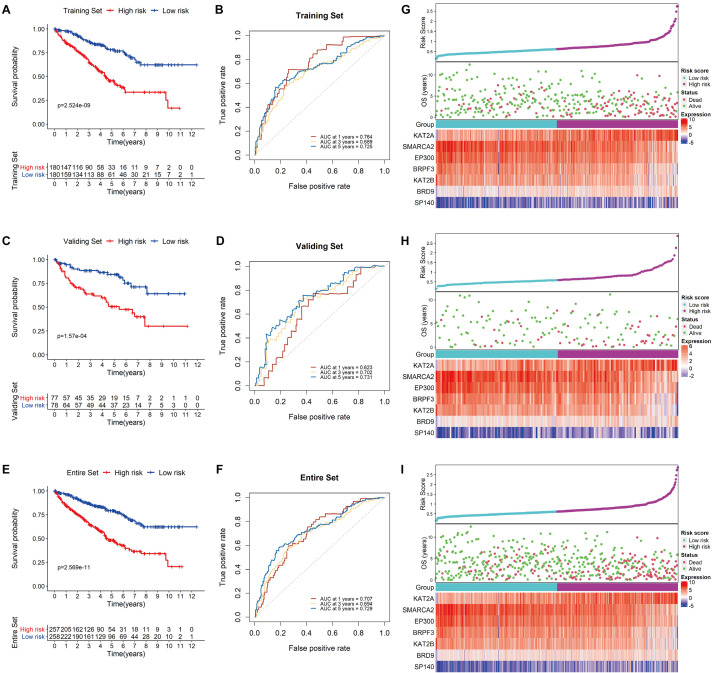
Validation of the signature. Kaplan-Meier survival analysis for low- and high-risk KIRC patients from TCGA based on our signature in the training set **(A)**, the validation set **(C)**, and the entire set **(E)**; prediction of recurrence risk in the training set at 1−, 3− and 5-year periods with BRD gene signature ROC analysis **(B)**, the validation set **(D)**, and the entire set **(F)**; the distribution of risk scores, OS and the final status and the expression patterns of the prognostic BRD genes in the training set **(G)**, the validation set **(H)**, and the entire set **(I)**.

Using the median risk score as a cutoff value, KIRC patients in the testing set were further divided into low-risk and high-risk groups. Survival analysis of the group was conducted, and the results showed that the survival rate of the patients in the low-risk group was considerably higher than that of those in the high-risk group (*P* < 0.0001; Figure 5A). In addition, the AUC values of 1−, 3− and 5-year OS were 0.764, 0.689 and 0.725, respectively, demonstrating the sensitivity and stability of the model (Figure 5B). Similar results were observed in both the validation and complete TCGA data sets (Figures 5C–F). The relationships among the risk scores, OS and corresponding BRD gene expression profiles are shown in Figures 5G–I. Taken together, these results demonstrated that the BRD gene signature used in this study worked well to predict the disease course of patients with KIRC.

### Construction and Evaluation of the Nomogram

**FIGURE 6:**
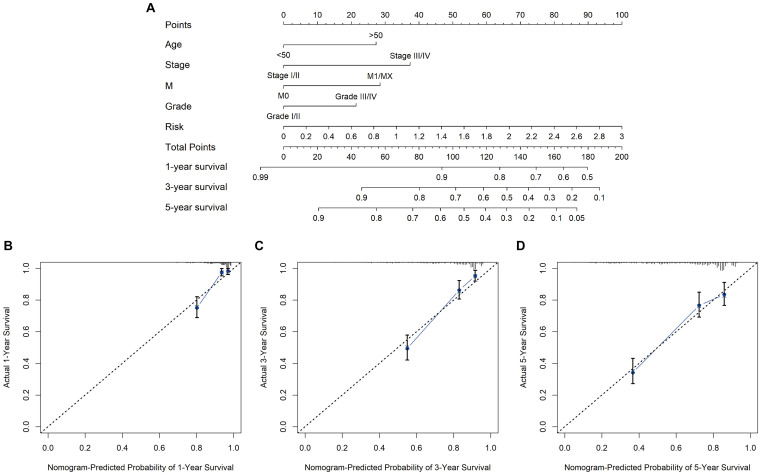
Nomogram and calibration plots. **(A)** 1−, 3−, and 5-year OS predicted by the nomogram. **(B)** Calibration curves for the prediction of 1-year OS. The probability of survival predicted by the nomogram plotted on the x-axis and actual survival on the y-axis. **(C)** Calibration curves for prediction of a 3-year OS. **(D)** Calibration curves for prediction of a 5-year OS.

The nomogram was constructed based on the following significant independent risk factors from univariate and multivariate analysis of Cox regression (Table 2): age (> 50 vs < 50), AJCC stage (stages I-II vs. III-IV), tumor grade (grade I-II vs. III-IV) and risk score (Figure 6A). The calibration plot indicated that the nomogram performed well in predicting the probability of 1−, 3− and 5-year OS in KIRC patients (Figures 6B–D).

**TABLE 2:** Univariable and multivariable Cox regression analyses of clinical characteristics.

Variables	Univariate				Multivariate			
	*P*-value	HR	HR.95L	HR.95H	*P*-value	HR	HR.95L	HR.95H
Age (> 50/ < 50)	< 0.001	2.229	1.398	3.554	0.01	1.856	1.158	2.974
Gender (female/male)	0.79	1.043	0.764	1.425	−			
Clinical stage (III–IV/I–II)	< 0.001	3.734	2.716	5.134	0.002	2.871	1.465	5.624
M (M1–MX/M0)	<	3.701	2.712	5.05	0.001	1.804	1.263	2.575
N (N1–NX/N0)	0.507	0.903	0.669	1.22	−			
T (T3–T4/T1–T2)	<0.001	3.042	2.242	4.127	0.419	0.78	0.426	1.427
Grade (G3–G4/G1–G2)	<0.001	2.643	1.875	3.726	0.011	1.611	1.116	2.326
Risk score	<0.001	2.553	1.977	3.296	< 0.001	2.07	1.537	2.789

### Validation of the Signature Using the HPA and GEO Databases

**FIGURE 7:**
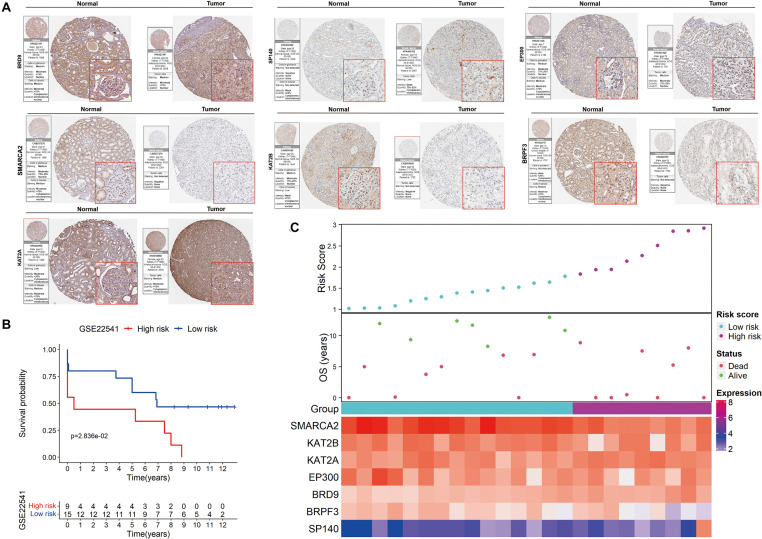
Validation of the signature on HPA and GEO. **(A)** The expression of the signature genes between KIRC and normal tissues at the translational level. **(B)** Kaplan-Meier survival analysis for low− and high-risk patients. **(C)** The distribution of risk scores, OS and the final status and the expression patterns of the signature genes.

The HPA database was also used to confirm the expression of the signature genes between renal clear cell carcinoma and normal tissues (Figure 7A). KAT2A, SP140 and BRD9 protein expression was higher in tumor tissues, and SMARCA2, BRPF3, KAT2B and EP300 protein expression was lower in the tumor tissues. These findings were consistent with our results in TCGA.

The prognostic value of the signature was further evaluated in the GSE22541 dataset, which contains complete transcriptome and clinical information of 24 KIRC patients. The risk score for each patient was calculated following the same formula. Patients were subsequently categorized into high- and low-risk groups based on the cutoff value of the risk score in TCGA. Survival analysis confirmed a lower survival rate in the high-risk group (Figure 7B). All patients in the high-risk group had disease progression, whereas all patients without disease progression were classified into the low-risk group (Figure 7C).

### Pan-Cancer Analysis of the BRD Genes of the Signature

**FIGURE 8:**
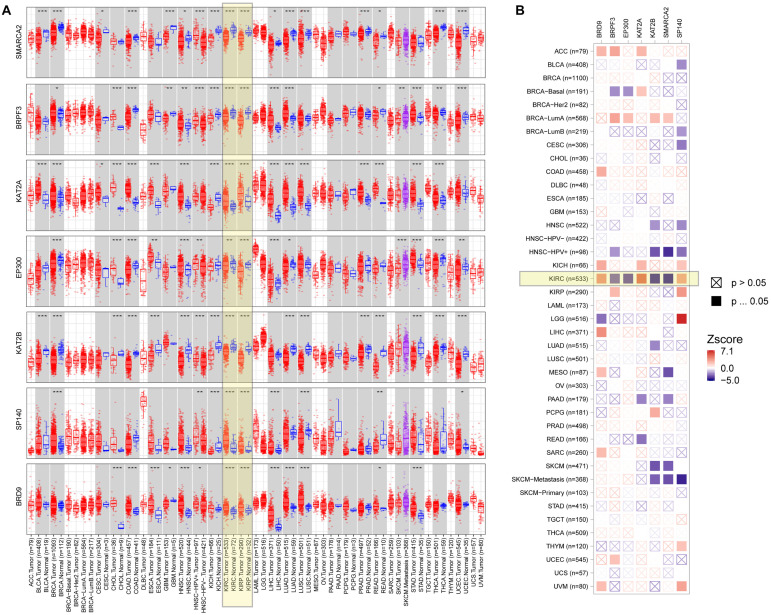
Expression level and clinical relevance of the signature genes in different tumors. **(A)** The expression status of the signature genes in different cancers or specific cancer subtypes (*P < 0.05; **P < 0.01; ***P < 0.001). **(B)** Heatmap table of the normalized coefficients of the signature gene expression in the Cox proportional hazard model.

To explore the role of seven BRD genes of the signature in cancers, we comprehensively evaluated their expression patterns and clinical impacts among pan-cancer samples (Figure 8). In the differential expression comparisons of tumor and normal tissues across different cancer types, these seven genes were all significantly (*P* < 0.01) differentially expressed in KIRC and KIRP samples (Figure 8A). Furthermore, the expression patterns of these genes in KIRC and KIRP samples were similar. Compared with other cancers, these seven genes were significantly associated with patient survival in KIRC (Figure 8B). Interestingly, a high expression level of the BRPF3 gene was associated with improved survival in KIRC, but the opposite results were noted for KIRP. The results indicated that the regulation pattern of these seven BRD genes might be specific to KIRC.

### GO, KEGG, and GSEA Analyses

**FIGURE 9:**
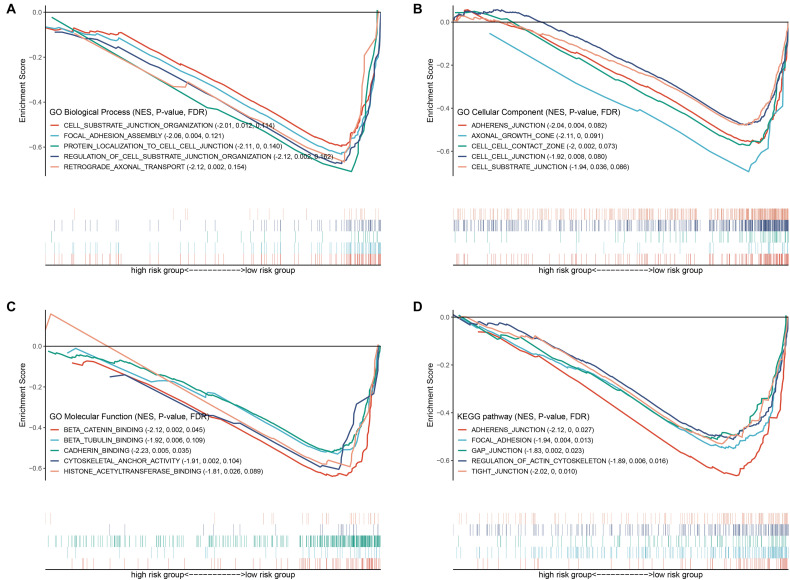
GSEA based GO and KEGG analysis. Gene sets of **(A)** GO biological process, **(B)** GO cellular component, **(C)** GO molecular function and **(D)** KEGG associated with the signature.

To explore the mechanisms underlying the BRD gene signature, we subsequently conducted biological process and pathway analysis using GSEA. The results demonstrated that “protein localization,” “axonal growth cone” and “beta catenin binding” were the most enriched terms in BP, MF and CC, respectively (Figures 9A–C). “Histone acetyltransferase binging” was enriched in GO MF as well. KEGG-based pathway enrichment analysis of these BRD genes showed the highest enrichment in “adherens junction,” “focal adhesion,” “gap junction,” “regulation of actin cytoskeleton” and “tight junction” (Figure 9D). These results clarified that these BRD genes in the low-risk group were involved in cell junctions, which are markers of epithelial features, and indicated that the high-risk group was more likely to exhibit metastasis.

## Discussion

Bromodomain genes have been reported to be carcinogenic or tumor suppressing in different cancer contexts, but the role of these genes in KIRC has rarely been reported. Utilizing global The Cancer Genome Atlas (TCGA) sequencing data, we were able to investigate the genetic status of a complete set of BRD genes. In our study, we identified seven BRD genes that were used to predict poor survival in patients with clear cell carcinoma of the kidney regardless of the treatment option. In other kidney cancer subtypes, some of these genes are also associated with patient prognosis.

In this study, we observed that seven BRD genes (*KAT2A, BRD9, SP140, EP300, SMARCA2, KAT2B*, and *BRPF3*) can be utilized as new targets for KIRC. *KAT2A*, *SP140*, and *BRD9* were highly expressed, whereas *SMARCA2*, *KAT2B*, *EP300*, and *BRPF3* were expressed at low levels in KIRC. KAT2B belongs to the GNAT (GCN5-related N-acetyltransferase) family, one of the five acetyltransferase families (Sheikh, 2014; Ganai et al., 2016). We also found a common deletion of *KAT2B* in KIRC, suggesting that the absence of *KAT2B* may be carcinogenic in KIRC. The role of *KAT2B* in cancer depends on the cancer background. At present, most studies tend to support the tumor suppressive effect of KAT2B, which is downregulated in esophageal squamous cell carcinoma (ESCC) and intestinal-type gastric cancer compared to adjacent non-tumor tissues (Zhu et al., 2009; Ying et al., 2010). In contrast, KAT2A protein was overexpressed and positively correlated with tumor size in human glioma and non-small-cell lung carcinoma (NSCLC) (Bondy-Chorney et al., 2019). KAT2A is also highly expressed in human pancreatic ductal adenocarcinoma (PDAC) specimens and positively correlated with advanced stages of PDAC and short patient survival (Tong et al., 2020). The results of this work are consistent with these reports. KAT2A, a histone acetyltransferase (HAT) and a member of the GCN5-related N-acetyltransferase (GNAT) superfamily, interacts with acetyl-CoA and transfers its acetyl residues to histones (Grant et al., 1997; Wang and Dent, 2014) and is associated with gene expression, tumor formation, and tumor cell proliferation (Wang et al., 2017).

As a histone acetyltransferase, EP300 plays an important role in cell proliferation and differentiation (Dancy and Cole, 2015). EP300 promotes acute lymphoblastic leukemia by regulating TCF3 HLF (American Association for Cancer Research, 2020). Through mouse genetics and human tumor sequencing analyses, EP300 has been widely associated with cancers and other pathological conditions (Attar and Kurdistani, 2017). In the present large cohort of large B-cell lymphoma patients, EP300 was highly recurrent and significantly associated with poor prognosis. EP300 expression also contributes to the growth of prostate cancer and is a predictor of aggressive characteristics of this cancer (Debes et al., 2003). Previous studies suggest that high expression of the transcriptional coactivator EP300 correlates with aggressive features of hepatocellular carcinoma and cutaneous squamous cell carcinoma (Li et al., 2011; Chen et al., 2015). Despite the cancer-promoting effects of EP300, multiple lines of evidence suggest that EP300 may also be involved in cancer suppression. The early indications for P300 to inhibit tumors were derived from a rare congenital developmental disorder, Rubinstein-Taybi Syn Drome (RTS) (Miller and Rubinstein, 1995). A study by Gayther et al. (2000) identified the first cancer-associated inactivating genetic lesions in EP300 in breast and colorectal primary tumors and cell lines. Our analysis showed that EP300 was expressed at low levels in KIRC. Many functions of EP300 may vary in cancers depending on the context, cellular identity, and perhaps environmental condition.

SMARCA2, is an adenosine triphosphatase (ATPase) (Phelan et al., 1999; Wilsker et al., 2004). The failure of various functions of the SWI/SNF complex can be identified in different parts of the tumor (Agaimy et al., 2016a, b). We found that the loss of the core protein SMARCA2 of the complex occurred in a subgroup of undifferentiated KIRC carcinomas. SP140 is a member of Group VII of the BRD family. These proteins exhibited high sequence similarities with autoimmune regulators (AIREs), but their molecular functions have not been elucidated (Gibson et al., 1998). The expression of immune-related genes associated with multiple sclerosis (MS) and other autoimmune diseases is regulated by SP140 (Karaky et al., 2018). We observed that *SP140* is upregulated in KIRC. BRPF3 binds to diacetylated and dipropylated histone ligands, but the affinity of their interactions has not been determined. We observed that *BRPF3* is downregulated in KIRC. The biological function of BRD9 is also unknown, but it has been shown to be related to a number of different cancer types. Previous studies have found that BRD9 is upregulated in ovarian cancer, and depleting BRD9 makes cancer cells more sensitive to olaparib and cisplatin (Zhou et al., 2020).

Among the 336 KIRC cases with sequencing data, mutations of BRD genes were identified in 190 independent samples, and the predominant alteration was characterized by CNV. The PBRM1 gene had the highest mutation frequency (45.83%, 154/336), followed by *SMARCA* (2.68%, 9/336). In primary and advanced RCC, the expression characteristics of cancer cell subsets and immune escape are associated with PBRM1 mutations (Bi et al., 2021).

In conclusion, in this work, we suggested a seven-gene risk profile (*KAT2A, BRD9, SP140, EP300, SMARCA2, KAT2B* and *BRPF3*) to predict the prognosis of KIRC patients. This gene signature provides a potential target for clinical treatment and diagnosis. Of course, the model still needs to be validated in prospective clinical trials with large sample sizes in the future.

## Data Availability Statement

Publicly available datasets were analyzed in this study. This data can be found here: TCGA (https://tcga-data.nci.nih.gov/).

## Ethics Statement

The studies involving human participants were reviewed and approved by The Cancer Genome Atlas. Written informed consent for participation was not required for this study in accordance with the national legislation and the institutional requirements.

## Author Contributions

JL, CQ, and YJ performed the online data mining and bioinformatic analysis. JL, CQ, and QB collectively wrote the manuscript. BL and QB designed the work. All authors read and approved the final manuscript.

## Conflict of Interest

The authors declare that the research was conducted in the absence of any commercial or financial relationships that could be construed as a potential conflict of interest.

## Footnotes

1 https://tcga-data.nci.nih.gov/

2 http://www.proteinatlas.org/

3 http://software.broadinstitute.org/gsea/index.jsp
